# Thiophenyl Anilato-Based NIR-Emitting Lanthanide (Ln^III^ = Er, Yb) Dinuclear Complexes

**DOI:** 10.3390/molecules29235804

**Published:** 2024-12-09

**Authors:** Fabio Manna, Mariangela Oggianu, Valentina Mameli, Stefano Lai, Angelica Simbula, Francesco Quochi, Narcis Avarvari, Maria Laura Mercuri

**Affiliations:** 1 Dipartimento di Scienze Chimiche e Geologiche, Università di Cagliari, Complesso Universitario di Monserrato, S.P. 8 Km 0.700, I-09042 Monserrato, Italy; mariangela.oggianu@unica.it (M.O.); valentina.mameli@unica.it (V.M.); mercuri@unica.it (M.L.M.); 2 Dipartimento di Fisica, Università di Cagliari, Complesso Universitario di Monserrato, S.P. 8 Km 0.700, I-09042 Monserrato, Italy; stefano.lai@dsf.unica.it (S.L.); asimbula@dsf.unica.it (A.S.); 3 Laboratoire MOLTECH-Anjou UMR 6200, UFR Sciences, CNRS, Université d’Angers, Bât. K, 2 Bd. Lavoisier, I-49045 Angers, France; narcis.avarvari@univ-angers.fr

**Keywords:** anilates, antenna effect, lanthanides, NIR emission, vibrational quenching

## Abstract

By combining Er^III^ and Yb^III^ ions with 3,6-dithiophene-anilate (Th_2_An) and scorpionate hydrotris(pyrazol-1-yl)borate (HBpz_3_^−^) ligands new luminescent dinuclear complexes are obtained. The two materials formulated as [((HB(pz)_3_)_2_Yb)_2_(μ-th_2_An)]·4DCM·1.3H_2_O **1Yb** and [((HB(pz)_3_)_2_Er)_2_(μ-th_2_An)]·4DCM·1.8H_2_O **1Er,** respectively, have been structurally characterized by SC-XRD and PXRD studies. This study presents a comprehensive investigation of the photophysical properties of the Th_2_An ligand for the first time. Our findings reveal the crucial role of the thiophene anilate as an effective optical antenna, which sensitizes near-infrared (NIR)-emitting lanthanide ions, specifically Er^III^ and Yb^III^. The significant impact of vibrational quenching on the Ln^III^ NIR emission efficiency has been also highlighted.

## 1. Introduction

Over several decades, lanthanide complexes have been largely investigated thanks to their intriguing structural topologies, and magnetic and optical properties that make them appealing in various fields, ranging from optoelectronics and bioimaging to data storage, molecular spintronics and electronics and telecommunications. Furthermore, the prediction of their crystal structure still remains a challenge due to the flexibility in the geometries and the high coordination numbers of the lanthanide (Ln^III^) ions. In this context, the synthetic conditions, the presence of coordinated solvent molecules, the choice of the organic linker and the nature of the Ln^III^ ion, are crucial aspects in the design of materials with luminescent properties.

Optical properties of lanthanides originate from their electronic configuration [Xe] 4f ^n^ (n = 0 to 14). Given the forbidden f-f transitions, Ln^III^ ions are characterized by very low molar absorption coefficients and consequently low emission properties, if directly excited [1]. The selection of organic linkers which can act as *antennas* for sensitizing Ln^III^ ions and the proper evaluation of the coordination modes of these linkers [2] is fundamental to obtaining luminescent complexes. Among various organic linkers, quinone derivatives and in particular, 3,6-disubstituted-2,5-dihydroxy-1,4-benzoquinones, commonly known as anilates (An), have been widely investigated thanks to (*i)* their Janus-type capability of coordinating different metal ions on two opposite sides of the planar benzoquinone *core* and (*ii)* the possibility to tune their chemical structure by changing the substituents at the 3,6 positions [3,4].

In 2015, Mercuri, Avarvari et al. [5] reported on the synthesis of the first heterosubstituted anilate, the chlorocyananilate ligand (3-chloro-6-cyano-2,5- dihydroxybenzoquinone, ClCNAn), which was largely studied as an antenna toward visible (Eu^III^ and Tb^III^) and near-IR (NIR, for Nd^III^, Er^III^ and Yb^III^) emitters Ln^III^ ions, to construct innovative materials as 2D coordination polymers (CPs), 2D metal–organic Nanosheets (MONs) [6,7,8,9] and 3D metal–organic frameworks (MOFs) [10].

Particularly, chlorocyananilate ligand acts as an efficient antenna toward Ln^III^ ions, by a multistep relaxation process involving, in sequence, intersystem crossing and energy transfer from ligand triplet states to the Ln^III^ ions. The whole mechanism has recently been elucidated by some of us through ab initio calculations for 2D CPs based on Er^III^ and Yb^III^ [8].

Recently, when substituting the 3,6 positions with triazole groups, some of us synthesized and fully characterized novel multifunctional 3D flexible MOFs based on 3,6-N-ditriazolyl-2,5-dihydroxy-1,4-benzoquinone (trz_2_An) [11] linker and Er^III^, Tb^III^, Ho^III^ and Dy^III^ ions showing a combination of luminescence and Single Ion Magnets (SIMs) properties. In particular, in the Er^III^-based MOFs, the structural flexibility is related to a sizeable change in the emission properties [12].

Interestingly, by combining Tb^III^, Er^III^ and Yb^III^ ions with the 3,6-dichloroanilate (Cl_2_An^2−^) ligand and the scorpionate hydrotris(pyrazol-1-yl)borate (HB(pz)_3_^−^) as capping ligand, dinuclear complexes have been obtained, showing luminescence properties, which confirm the efficient antenna effect of the anilate derivative toward NIR emitting Ln^III^ ions [13]. It is worth saying that, to the best of our knowledge, the only anilates that have proven to be efficient antennas toward NIR emitting Ln^III^ ions are the three mentioned above (ClCNAn^2−^, trz_2_An^2−^ and Cl_2_An^2−^). Furthermore, only ClCNAn^2−^ and trz_2_An^2−^ show ligand-centered emission in the visible range, although, thanks to their strong absorption in the visible range, this class of molecules is particularly suitable for energy transfer toward NIR emitting Ln^III^ ions, according to the empirical Lathva’s rule [14].

Very recently, some of us reported on the synthesis, structural and magnetic characterization of novel dinuclear Dy^III^ complexes based on [((HB(pz)_3_)_2_Dy)_2_(μ-Th_2_An)] (**1Dy**) (Th_2_An^2−^ = 2,5-Dihydroxy-3,6-bis(thiophene-2-yl)cyclohexa-2,5-diene-1,4-dione) and [((HB(pz)_3_)_2_Dy)_2_(μ-ClCNAn)] (**2Dy**) (ClCNAn^2−^ = 3-chloro-6-cyano-anilate) [15], showing field-induced SMM properties. 3,6-dithiophene-anilate (Th_2_An) has been synthesized in 2014 by Mercuri, Avarvari et al. and it was used as a ligand to generate a mononuclear square planar heteroleptic Cu^II^ complex [16]. Despite the strong adsorption properties due to the charge transfer between thiophene rings, at 3,6 positions and the benzoquinone *core* [15,16], its potential as *an antenna* toward NIR emitting lanthanides was never investigated up to now. On these bases, we report herein the synthesis, structural characterization and luminescent properties studies of two lanthanide dinuclear complexes, **1Er** and **1Yb**, obtained by changing Dy^III^ ions, in **1Dy** selected as ideal platform, with Er^III^ and Yb^III^ ions.

## 2. Results and Discussion

### 2.1. Synthesis and Characterization

**Scheme 1:**
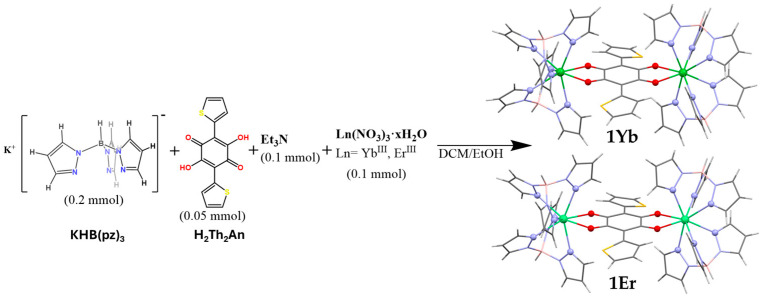
Synthesis of complexes **1Yb** and **1Er**.

Compounds **1Yb** and **1Er** were synthesized, as reported in Scheme 1, using the protocol reported by some of us for the isostructural Dy^III^ dinuclear complex [15]. Briefly, a one-pot reaction is carried out in a dichloromethane (DCM)/ethanol (EtOH) mixture. In particular, first a solution of the capping ligand HBpz_3_^−^ is added to a solution of the salt Ln(NO_3_)_3_ and later a solution of the anilate bridging ligand, deprotonated with triethylamine (Et_3_N), with a 4:2:1 molar ratio, respectively. The solution is kept at room temperature, leading to the formation, in both cases, of pure green prismatic crystals of the dinuclear complex suitable for single-crystal X-ray diffraction (SCXRD). A multi-technique approach as that reported for the Dy^III^ isostructural complex was used to characterize and confirm the purity of **1Yb** and **1Er** (SCXRD, PXRD, IR, UV-vis spectroscopy, mass spectrometry and elemental analysis). The spectroscopic characterizations match with those reported for the Dy^III^ compound with slight shifts in the IR (Figure S1) and UV-Vis spectra (*vide infra*). The shifts in the IR bands are attributed to the different sizes of the Ln^III^ involved in the complex formation, similar to what was reported for the dinuclear chloroanilate series. ESI-Mass Spectrometry confirmed the presence of the compound in the solution and PXRD (Figure S2) was performed on the freshly prepared sample to confirm the phase purity of the bulk and to avoid crystallization solvent loss (*vide infra*) before UV-Vis-NIR spectroscopy characterization.

### 2.2. Crystal Structures

**Figure 1:**
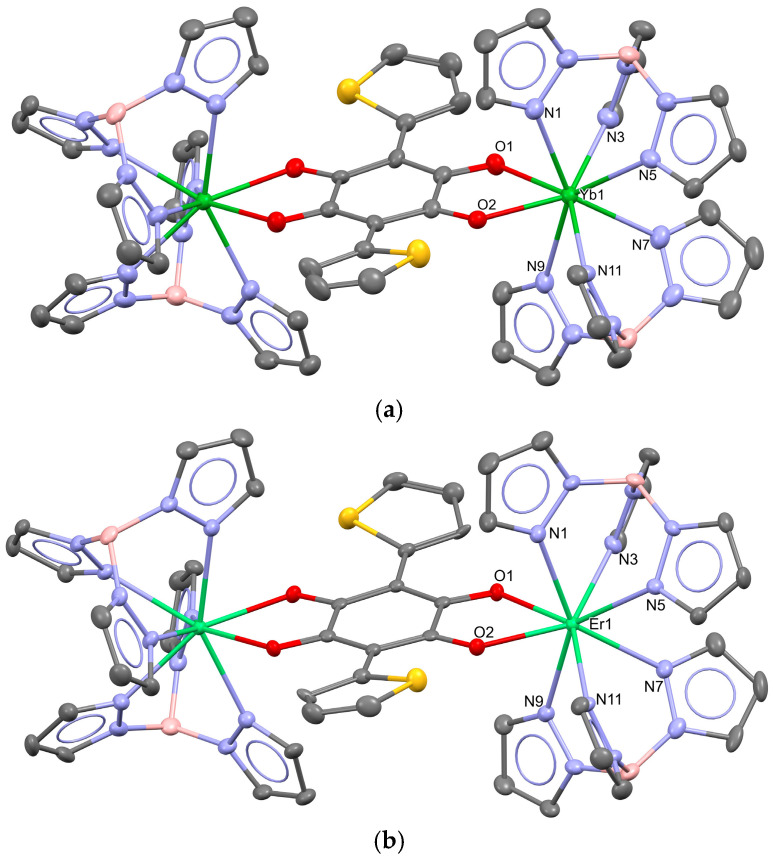
Structural representations of the dinuclear complexes **1Yb** (**a**) and **1Er** (**b**) with thermal ellipsoids drawn at a 50% probability level. Hydrogen atoms and co-crystallized DCM molecules have been omitted for clarity. Color code: C (grey), N (blue), O (red), S (yellow), B (pink), Yb (green) and Er (light green).

**1Yb** and **1Er** crystallize in the tetragonal P4_2_/n space group, the crystal structure of the dinuclear complexes being shown in Figure 1.

The asymmetric unit is composed of one Ln^III^ ion, two molecules of HB(pz)_3_^−^, half molecule of the Th_2_An^2−^ ligand and two molecules of DCM as co-crystallization solvent. Furthermore, we modeled the residual electron density inside the voids of the structure by solvent mask procedure (Olex2) [17], as for the Dy compound, and 1.3 and 1.8 water molecules per formula unit were determined for **1Yb**, formulated as [((HB(pz)_3_)_2_Yb)_2_(μ-th_2_An)]·4DCM·1.3H_2_O, and **1Er**, formulated as [((HB(pz)_3_)_2_Er)_2_(μ-th_2_An)]·4DCM·1.8H_2_O, respectively. Two molecules of the capping HBpz_3_^−^ ligand coordinate the Ln^III^ by the three N atoms of each pyrazole ring, and these entities are bridged by the anilate ligand which coordinates each Ln^III^ ion by the two oxygens of the benzoquinone moiety in a bidentate manner allowing the formation of the dinuclear neutral complex. Consequently, considering the centrosymmetric nature of the complex, each Ln^III^ ion is eight-coordinated with a {N_6_O_2_} coordination sphere. Crystal packing is the same as the Dy compound due to isostructurality. The differences in cell parameters, structural void occupation and the bond distances are attributed to lanthanide contraction (the selected distances are reported in Table 1). Indeed, the coordination bonds Yb-O and Yb-N are shorter (2.307, 2.317 Yb-O and between 2.370–2.478 Yb-N) than the Er-O and Er-N ones (2.327, 2.343 and between 2.490–2.405 Er-N) similarly to those reported for analogous of chloranilate-bridged dinuclear complexes. Interestingly, the coordination around the Ln^III^ ion remains almost unchanged within the series contrary to what is reported for the analogous [18,19] compounds. In fact, the deviation values from the ideal coordination geometries calculated with Shape2.1 [20] are very similar to those obtained for the Dy^III^ compound allowing to assign a SAPR-8 coordination in all cases. As reported for the Dy^III^ complex, the deviation values are not negligible, highlighting a distortion from ideal geometry. Furthermore, the values are close to those calculated for biaugmented trigonal prism in *C*_2v_ coordination geometry types, showing similar distortions and coordination within the series. All these results are summarized in Table 1.

It is well known that the luminescence efficiency of Ln^III^ ions, particularly in the NIR region, is affected by the presence of quenching groups around the emitting ion [21,22,23,24,25,26]. According to theory, quenching occurs through a resonant mechanism between the vibrations of the functional groups present in proximity and the energy levels of the Ln^III^ ions. Consequently, functional groups with high vibrational energies such as (C-H, N-H and O-H) are particularly efficient in the quenching of NIR-emitting Ln^III^ ions, since their lower overtones fall close to the Ln^III^ emission frequencies [21,22,23]. Therefore, the presence of quenching groups and their distances from the Ln^III^ ions are crucial parameters for the emission efficiencies. In our case, there are several high-vibration groups [24] present in the crystal structure such as aromatic C-H, aliphatic C-H and B-H. Both intramolecular and intermolecular Ln^III^⋯H contact interactions are present, and the main ones are reported in Table 2.

All these interactions contribute to the quenching of Ln^III^ emission particularly the Ln^III^⋯H interactions in the first coordination sphere (distances smaller than 5 Å). The analysis of these Ln^III^⋯H contact distances shows the presence of several quenching groups within 5 Å suggesting a strong quenching effect on the Ln^III^ NIR emission [21]. Particularly, both complexes have contact distances in this range with the capping ligand: two with the B-H groups and six with the C-H at the 3-position of the pyrazole ring. The bridging ligand th_2_An shows only one of these interactions involving the C-H at the 3-position of the thiophene ring. The presence of nine different quenching groups in the first coordination sphere of Ln^III^ suggests a strong quenching effect in the solid state. Furthermore, in the solid state other intermolecular interactions are present that can add another contribution to the quenching mechanism. Three out of four C-H groups of the co-crystallized DCM molecules and one C-H group belonging to the pyrazole moiety of an adjacent complex are closer than 5 Å from the Ln^III^ ion. Although the distance of 5 Å is often used as a cut-off for the action of quenching groups, the second coordination sphere and therefore the quenching groups between 5 and 7.5 Å can lead to a non-negligible contribution [21]. In our case, given the molecular packing, a high number of these contact interactions are present (Figure S3) together with the presence of water-disordered molecules inside the void of the structure with O-H quenching groups. Therefore, another strong quenching contribution from the second coordination sphere on Ln^III^ emission is expected (vide infra) [22]. In fact, considering the strong bipolar moment of the O-H group, the overtones of this group have a strong absorbance. Furthermore, although they are disordered within the structure, they are located within voids close enough to quench the Ln^III^. Particularly, two voids are present in the unit cell with prolate shape (Figure S4) containing approximately three water molecules each (considering the solvent mask procedure), the centroids of these voids were calculated with Olex2 and they are at fractional coordinates of (¼, ¼, ¼) and (¾, ¾, ¾). The radius of the largest spherical void is 2.0 Å in both cases. Considering that the centroid is about 8 Å from the nearest Ln^III^ we can estimate that the H atoms of the water molecules inside the voids could approach the metals as close as 6 Å. Furthermore, since more than one water molecule is present in one void we cannot exclude that the hydrogen atoms may be located at the edge of the contact surface area (Figure S4) that, in the region near the scorpionate ligand, approach the Ln^III^ with distances around 5 Å.

### 2.3. UV-Vis-NIR Spectroscopy

**Figure 2:**
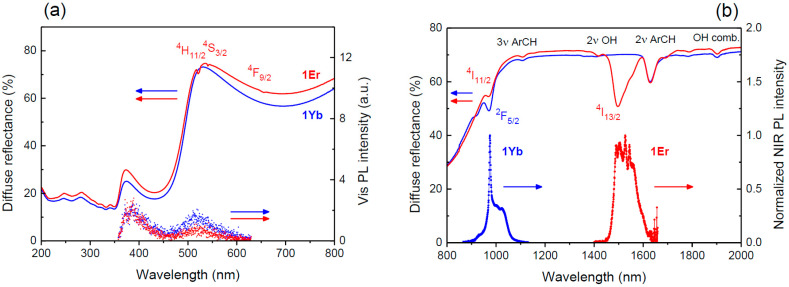
Diffuse reflectance and emission spectra of Ln^III^ dinuclear complexes in the solid state. (**a**) UV-Vis range, showing ligand-centered absorption and emission (excitation at 350 nm). (**b**) NIR range, highlighting Ln^III^-centered emission (excited at 355 nm) and vibrational absorption bands. Spectroscopic terms for the Ln^III^ upper manifolds, as well as key vibrational overtones and combination modes, are labeled. Diffuse reflectance spectra are represented by solid lines, while PL spectra are shown as dotted lines in both panels.

The optical absorption of the Ln^III^ complexes in the solid state within the UV-Vis range, presented in Figure 2a (left axis) as diffuse reflectance spectra, is primarily attributed to the thiophenyl anilate ligand (Th_2_An^2−^). Band assignments for these spectra have been previously reported in detail [15]. Comparative measurements in dilute ACN solution confirmed that the HB(pz)_3_^−^ ligand contributes negligibly to the absorption (Figure S5a).

Due to the heavy-atom effect, which promotes intersystem crossing and quenches singlet emission, visible photoluminescence (Vis PL) from ligand-centered transitions was not detectable under continuous-wave (cw) excitation at low irradiance levels. Instead, Vis PL spectra were collected under ultrafast pulsed excitation, revealing weak ligand-centered PL that exhibited a pronounced inner filter effect caused by the Th_2_An^2−^ absorption band peaking at 450 nm (Figure 2a, right axis).

The near-infrared (NIR) emission of Yb^III^ and Er^III^ is effectively sensitized under UV excitation, thanks to the antenna effect facilitated by the Th_2_An^2−^ ligand [8,27]. Figure 2b displays the NIR spectra corresponding to the Yb^III^ (^2^F_5/2_ → ^2^F_7/2_) and Er^III^ (^4^I_13/2_ → ^4^I_15/2_) transitions, observed at approximately 1 μm and 1.5 μm, respectively. A comparison between diffuse reflectance and PL spectra in the NIR region reveals a clear correspondence between the Ln^III^ absorption and emission bands in both **1Yb** and **1Er**. Additionally, several absorption bands were detected and attributed to vibrational overtones: the first (2ν) and second (3ν) overtones of the aromatic C–H (ArCH) stretching mode, the first overtone of the O–H stretching mode, and a combination mode of O–H vibrations in water, in agreement with what was found in the crystal structure.

**Figure 3:**
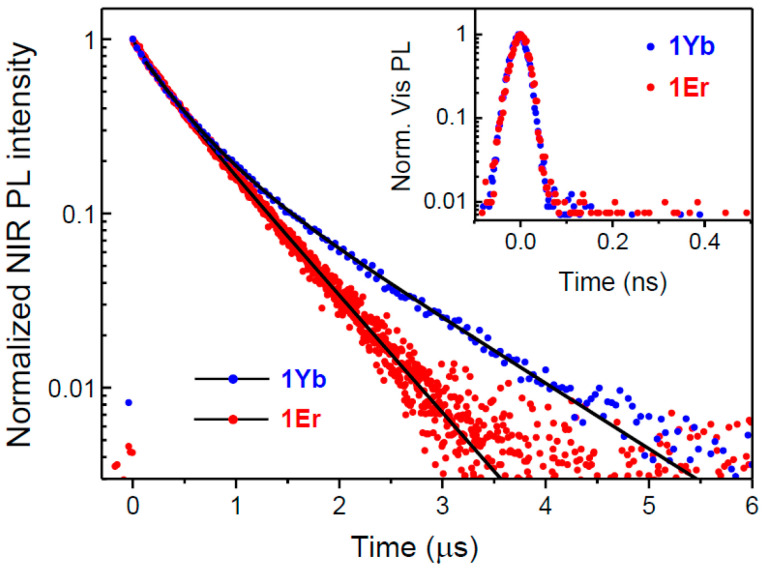
Time-resolved NIR PL of Ln^III^ dinuclear complexes in the solid state (excitation at 355 nm). Inset: Time-resolved and spectrally integrated Vis PL of Ln^III^ complexes in the solid state (excitation at 350 nm). Dots: Experimental data; Black lines: Biexponential decay fits; refer to the text for details.

The Ln^III^-centered NIR emission, sensitized by UV pulsed excitation, exhibits decay dynamics on the microsecond timescale for both **1Yb** and **1Er** (Figure 3). Amplitude-averaged decay time constants, derived from biexponential fits, are approximately 0.7 µs for **1Yb** and 0.6 µs for **1Er**, respectively. This behavior is attributed to vibrational quenching [28], primarily caused by the aromatic C–H groups in the ligands and the O–H groups of water molecules. These water molecules, which occupy voids within the crystal structure, can approach the Ln^III^ centers at distances of approximately 5 Å. Notably, the second overtone (3ν) of the O–H stretching mode in water, which is closely resonant with the emission spectrum of the Yb^III 2^F_5/2_ manifold and consequently remains spectrally unresolved in absorption, is inferred to significantly reduce the emission efficiency of the Yb^III^ ions, as evidenced by the observed submicrosecond emission lifetime.

As shown in Figure S5b, the time-resolved Vis PL of ligand species in dilute ACN solutions under pulsed UV irradiation exhibits a monoexponential decay with a characteristic lifetime of 0.9 ns for HB(pz)_3_^−^. In contrast, H_2_Th_2_An displays a nearly biexponential decay with characteristic lifetimes of 1.0 ns and 6.0 ns, suggesting the presence of a potential equilibrium between two protonation states. In the complexes, where the Th_2_An^2−^ and HB(pz)_3_^−^ ligands are coordinated to the Ln^III^ ions, the significant heavy-atom effect promotes rapid intersystem crossing, effectively depleting the ligands’ singlet states and leading to ultrafast Vis-PL decay. As a result, the time-resolved and spectrally integrated Vis PL becomes resolution-limited (see inset of Figure 3) for both **1Yb** and **1Er**.

## 3. Materials and Methods

### 3.1. General Procedure

All chemicals purchased (Merck, Darmstadt, Germany) were of reagent grade or higher and used as received. 2,5-Dihydroxy-3,6-bis(thiophene-2-yl)cyclohexa-2,5-diene-1,4-dione has been synthesized as previously reported [15]. Thermo Fisher Orbitrap Elite spectrometer (Thermo Fisher, Bothell, WA, USA) was used for electrospray ionization mass spectrometry (ESI-MS) exact mass determination. The IR spectra were performed on a JASCO ATR PRO 4X spectrophotometer (JASCO Corporation, Tokyo, Japan) in the range 4000–400 cm^−1^. Powder X-ray diffraction was performed using a θ–θ Bragg–Brentano geometry Seifert X 3000 diffractometer equipped with a Cu Kα source (λ = 1.5418 Å). Elemental analyses (C, H, N and S) were performed with a CE Instruments EA 1110 CHNS (CE Instruments Ltd., Wigan, UK).

### 3.2. Synthesis of [((HB(pz)_3_)_2_Yb)_2_(μ-th_2_An)]·4DCM·1.3H_2_O (1Yb) and [((HB(pz)_3_)_2_Er)_2_(μ-th_2_An)]·4DCM·1.8H_2_O (1Er)

The corresponding Ln(NO_3_)_3_·xH_2_O (0.1 mmol, Ln^III^ = Yb^III^, Er^III^) was dissolved in 5,5 mL of DCM/EtOH 10:1, KHB(pz)_3_ 50.5 mg (0.2 mmol) was dissolved in 3 mL of DCM/EtOH and added dropwise under stir affording a white precipitate. After 5 min of stirring, a blue solution of deprotonated th_2_An^2−^ ligand (15.2 mg of H_2_th_2_An, 0.05 mmol) dissolved in 5 mL of DCM, and 15 µL of Et_3_N as a base, was added dropwise affording a green solution with some white fine precipitate. The solution, after 10 min of stirring, was filtered through Celite and let to slowly evaporate at RT for two days. After slow evaporation green prismatic crystals were collected by filtration under vacuum (85% yield, 1Er; 82% 1Yb). Elemental analysis calc. for C_54_H_56.6_B_4_Cl_8_N_24_O_5.3_S_2_Yb_2_: C (34.80%), H (3.06%), N (18.04%), S (3.44%); found: C (34.83%), H (3.01%), N (18.05%), S (3.40%). calc. for C_54_H_57.6_B_4_Cl_8_Er_2_N_24_O_5.8_S_2_: C (34.85%), H (3.12%), N (18.06%), S (3.45%); found: C (34.88%), H (3.10%), N (18.03%), S (3.41%). ESI-MS **1Yb**: 1501.2812 ([M + H]^+^, C_50_H_47_B_4_N_24_O_4_S_2_Yb_2_; calc. 1501.2820). ESI-MS **1Er**: 1489.2706 ([M + H]^+^, C_50_H_47_B_4_N_24_O_4_S_2_Er_2_; calc. 1489.2691).

### 3.3. Structural Analysis of 1Yb and 1Er

Single crystals of **1Yb** and **1Er** were maintained in contact with the mother liquor and transferred to oil before SCXRD measurement. A suitable crystal was selected, and data were collected on a Bruker D8 Venture diffractometer equipped with a PHOTON II area detector. The crystal was kept at 100(2) K during data collection. Using Olex2, version 1.5 [17], the structure was solved with the SHELXT 2018/2 [29] structure solution program using Intrinsic Phasing and refined with the SHELXL 2018/3 [30] refinement package using least squares minimization. All non-hydrogen atoms were refined anisotropically. The hydrogen atoms were placed in calculated positions and refined isotropically with a riding model. The disordered water molecules inside the voids were modeled through a solvent mask implemented on Olex2 1.5. A summary of the crystallographic data and the structure refinement is given in Table 3. CCDC 2392147 and 2392152 contain the supplementary crystallographic data for **1Yb** and **1Er**, respectively. These data can be obtained free of charge via http://www.ccdc.cam.ac.uk/conts/retrieving.html (accessed on 18 October 2024) or from the CCDC, 12 Union Road, Cambridge CB2 1EZ, UK; Fax: +44 1223 336033; E-mail: deposit@ccdc.cam.ac.uk.

### 3.4. Optical Characterization

Diffuse reflectance spectra of Ln^III^ complexes in the solid state were recorded using a double-beam Agilent Cary 5000 UV-Vis-NIR spectrophotometer (Agilent Technologies, Santa Clara, CA, USA) equipped with an integrating sphere accessory. Finely ground crystals were used for measurements in the NIR range, while for the UV-Vis spectral range, the samples were dispersed in KBr at a 2 wt.% concentration to prevent absorption saturation of the ligand-centered transitions.

Molar absorptivity and cw Vis PL of the ligand species HB(pz)_3_^−^ and H_2_Th_2_An were measured in dilute ACN solutions using 10 mm thick quartz cuvettes at a concentration of 5 × 10^−^⁵ M. Molar absorptivity was determined using the same spectrophotometer employed for the diffuse reflectance measurements. Cw Vis PL spectra were recorded using a Horiba Fluoromax-4 fluorometer (Horiba, Kyoto, Japan), with excitation at 350 nm. The PL signal was passed through a GG400 Schott glass filter (Schott, Mainz, Germany) and corrected for both the instrument’s spectral response and the optical absorption of each sample.

Time-resolved Vis PL was measured using 100 fs pulses at 350 nm from a Light Conversion TOPAS 800 optical parametric amplifier (Light Conversion, Vilnius, Lithuania), pumped by a Coherent Libra Ti/sapphire regenerative amplifier (Coherent, Saxonburg, PA, USA), operating at a 1 kHz repetition rate. Dilute ACN solutions with concentrations of 5 × 10^−^⁴ M for ligand species were examined in 1 mm thick quartz cuvettes, alongside finely ground crystals of **1Yb** and **1Er**. The excitation fluence was carefully adjusted to ensure that all compounds remained within the linear response regime. The time-resolved Vis PL detection system comprised an Acton SpectraPro 2300i grating spectrometer (Acton Research, Acton, MA, USA) and a Hamamatsu C10910 streak camera (Hamamatsu Photonics, Hamamatsu City, Japan), yielding a temporal resolution of approximately 40 ps.

For time-resolved NIR PL measurements, samples were excited by a Teem Photonics PNV-M02510 Q-switched laser (Teem Photonics, Meylan, France), generating ~0.3 ns long pulses at 355 nm with a 1 kHz repetition rate. PL spectra were acquired by an Acton SpectraPro 2300i grating spectrometer (Acton Research, Acton, MA, USA) equipped with an Andor iDus InGaAs 1.7 µm array detector (Oxford Instruments Andor, Belfast, UK). For PL decay traces, detection was performed with a Hamamatsu H10330-75 InGaAs photomultiplier (Hamamatsu Photonics, Hamamatsu City, Japan), connected to a Tektronix TDS 5104 digital oscilloscope (Tektronix, Beaverton, OR, USA).

## 4. Conclusions

In conclusion, we successfully synthesized and characterized two novel members of the anilate-bridged dinuclear complex family, utilizing the dithiophene-anilate ligand Th_2_An, the trispyrazolylborate capping ligand and NIR-emitting Ln^III^ ions. These complexes are formulated as [((HB(pz)_3_)2Yb)2(μ-th_2_An)]·4DCM·1.3H_2_O (**1Yb**) and [((HB(pz)3)2Er)2(μ-th2An)]·4DCM·1.8H_2_O (**1Er**).

We investigated the photophysical properties of these complexes, which exhibit sensitized NIR emission upon UV excitation. This observation provides evidence for the antenna effect of the Th_2_An ligand in sensitizing Yb^III^ and Er^III^ ions. Our experimental results suggest that vibrational quenching mechanisms play a significant role, occurring within a few angstroms of the lanthanide centers. This quenching is attributed to the combined effects of the aromatic C–H groups of the ligands and the O–H groups of disordered water molecules that occupy the voids within the crystal structure.

Overall, this work emphasizes useful considerations for the design and development of new NIR-emitting Ln^III^ complexes based on the Th_2_An ligand. The insights gained from this study may pave the way for future advancements in the field of luminescent coordination materials.

## Figures and Tables

**Table 1:** Selected bond distances and continuous shape analysis for **1Yb** and **1Er**.

Selected Bond Distances (Å) 1Yb		Selected Bond Distances (Å) 1Er	
**M-O**			
Yb1-O1	2.307(4)	Er1-O1	2.327(2)
Yb1-O2	2.317(4)	Er1-O2	2.343(2)
**M-N**			
Yb1-N1	2.478(5)	Er1-N1	2.490(3)
Yb1-N3	2.370(5)	Er1-N3	2.405(3)
Yb1-N5	2.457(5)	Er1-N5	2.471(3)
Yb1-N7	2.467(5)	Er1-N7	2.484(3)
Yb1-N9	2.459(5)	Er1-N9	2.476(3)
Yb1-N11	2.403(5)	Er1-N11	2.426(3)
**M⋯M** Intramolecular			
Yb⋯Yb	8.5232(7)	Er⋯Er	8.5686(5)
**M⋯M** Intermolecular *			
Yb⋯Yb	8.0119(7)	Er⋯Er	8.0390(6)
CShM parameter (Shape 2.1) parameter			
	**1Yb**		**1Er**
**SAPR-8 *D*_4d_**	1.932		1.907
JBTPR-8 *C*_2v_	2.268		2.253
BTPR-8 *C*_2v_	2.279		2.276
SAPR-8 (square antiprism), BTPR-8 (biaugmented trigonal prism), JBTPR-8 (biaugmented trigonal prism J50).			

* Intermolecular lowest distances between two Ln^III^ of different complexes.

**Table 2:** Selected Ln^III^⋯H distances for **1Yb** end **1Er**.

intramolecular < 5 Å **1Yb**		intramolecular < 5 Å **1Er**	
Yb⋯H (B-H_pz_)		Er⋯(B-H_pz_)	
Yb⋯H1	4.569	Er⋯H1	4.593
Yb⋯H2	4.559	Er⋯H2	4.574
Yb---H (C-Hth)		Er⋯H (C-Hth)	
Yb⋯-H5	4.222	Er⋯H5	4.244
Yb⋯H (C-H_pz_)		Er⋯H (C-H_pz_)	
Yb⋯H23	3.592	Er⋯H20	3.703
Yb⋯H20	3.688	Er⋯H23	3.612
Yb⋯H8	3.699	Er⋯H8	3.707
Yb⋯H11	3.543	Er⋯H11	3.555
Yb⋯H14	3.648	Er⋯H14	3.647
Yb⋯H17	3.678	Er⋯H17	3.696
intermolecular < 5 Å **1Yb**		intermolecular < 5 Å **1Er**	
Yb⋯H (C-H_pz_)		Er⋯H (C-H_pz_)	
Yb⋯H16′′	4.188	Er′′′′⋯H16′′′′′	4.193
Yb⋯H (C-H_DCM_)		Er⋯H (C-H_DCM_)	
Yb⋯H26B′′′	4.046	Er′′′′′⋯H27B′′′′′′	4.073
Yb’⋯H27A	4.205	Er⋯H26B	4.228
Yb’⋯H27B	4.863	Er⋯H26A	4.888

Symmetry code: (′) 1/2 − x,1.5 − y,z; (′′)1/2 − y,x,1.5 − z; (′′′) − x,1 − y,1 − z; (′′′′)1/2 − y,−1 + x,1.5 − z; (′′′′′)1.5 − x,1/2 − y,z; (′′′′′′)1 − x,1 − y,1 − z.

**Table 3:** Crystal data and structure refinement for compounds **1Yb** and **1Er**.

	1Yb	1Er
Empirical formula	C_54_H_56.6_B_4_Cl_8_N_24_O_5.3_S_2_Yb_2_	C_54_H_57.6_B_4_Cl_8_Er_2_N_24_O_5.8_S_2_
Formula weight	1863.67	1861.12
Temperature/K	100.00	102.00
Crystal system	tetragonal	tetragonal
Space group	P4_2_/n	P4_2_/n
a/Å	17.4697(11)	17.5228(3)
b/Å	17.4697(11)	17.5228(3)
c/Å	23.331(2)	23.4310(4)
α/°	90	90
β/°	90	90
γ/°	90	90
Volume/Å^3^	7120.3(11)	7194.5(3)
Z	4	4
ρ_calc_g/cm^3^	1.739	1.718
μ/mm^−1^	3.034	2.737
F(000)	3676.0	3680.0
Crystal size/mm^3^	0.26 × 0.1 × 0.02	0.29 × 0.07 × 0.04
Radiation	MoKα (λ = 0.71073)	MoKα (λ = 0.71073)
2Θ range for data collection/°	3.73 to 56.644	3.718 to 56.574
Index ranges	−23 ≤ h ≤ 21, −23 ≤ k ≤ 23, −31 ≤ l ≤ 31	−22 ≤ h ≤ 23, −22 ≤ k ≤ 23, −31 ≤ l ≤ 31
Reflections collected	110554	91622
Independent reflections	8865 [R_int_ = 0.0940, R_sigma_ = 0.0417]	8933 [R_int_ = 0.1002, R_sigma_ = 0.0498]
Data/restraints/parameters	8865/0/442	8933/0/442
Goodness-of-fit on F^2^	1.057	1.033
Final R indexes [I ≥ 2σ (I)]	R_1_ = 0.0473, wR_2_ = 0.1342	R_1_ = 0.0331, wR_2_ = 0.0642
Final R indexes [all data]	R_1_ = 0.0808, wR_2_ = 0.1568	R_1_ = 0.0591, wR_2_ = 0.0709
Largest diff. peak/hole/e Å^−3^	1.94/−1.61	0.57/−0.65

## Supplementary Materials

The following supporting information can be downloaded at: https://www.mdpi.com/article/10.3390/molecules29235804/s1, Figure S1: ATR spectra of Ln^III^ complexes; Figure S2: PXRD pattern for **1Yb** and **1Er**; Figure S3: Representation of the intermolecular Ln^III^⋯H contact; Figure S4: Contact surface area representation of the voids obtained through Mercury software for **1Yb** (a) and **1Er** (b); Figure S5: Optical spectroscopy of ligand species in dilute ACN solutions.
