# Supplementary material for: Thiophenyl Anilato-Based NIR-Emitting Lanthanide (LnIII = Er, Yb) Dinuclear Complexes
Source: Molecules. 2024 Dec 9;29(23):5804. doi: 10.3390/molecules29235804 (PMC11643822; doi:10.3390/molecules29235804)
Supplement: Supplementary file 1 [file molecules-29-05804-s001.zip › molecules-3302545-supplementary.pdf]

molecules

MDPI

# Supporting Information

# Thiophenyl anilato-based NIR-emitting lanthanide ($Ln^{III}$ = Er, Yb) dinuclear complexes

**Fabio Manna [1*], Mariangela Oggianu [1], Valentina Mameli [1], Stefano Lai [2], Angelica Simbula [2], Francesco Quochi [2*], Narcis Avarvari [3] and Maria Laura Mercuri [1]**

[1] Dipartimento di Scienze Chimiche e Geologiche, Università di Cagliari, Complesso Universitario di Monserrato, S.P. 8 Km 0.700, I-09042 Monserrato (CA), Italy; mariangela.oggianu@unica.it (M.O.); valentina.mameli@unica.it (V.M.); mercuri@unica.it (M.L.M.)
[2] Dipartimento di Fisica, Università di Cagliari, Complesso Universitario di Monserrato, S.P. 8 Km 0.700, I-09042 Monserrato (CA), Italy; stefano.lai@dsf.unica.it (S.L.); asimbula@dsf.unica.it (A.S.)
[3] Univ Angers, CNRS, MOLTECH-Anjou, SFR MATRIX, F-49000 Angers, France; Narcis.Avarvari@univ-angers.fr (N.A.)

* Correspondence: quochi@unica.it (F.Q.); fabio.manna@unica.it (F.M.)

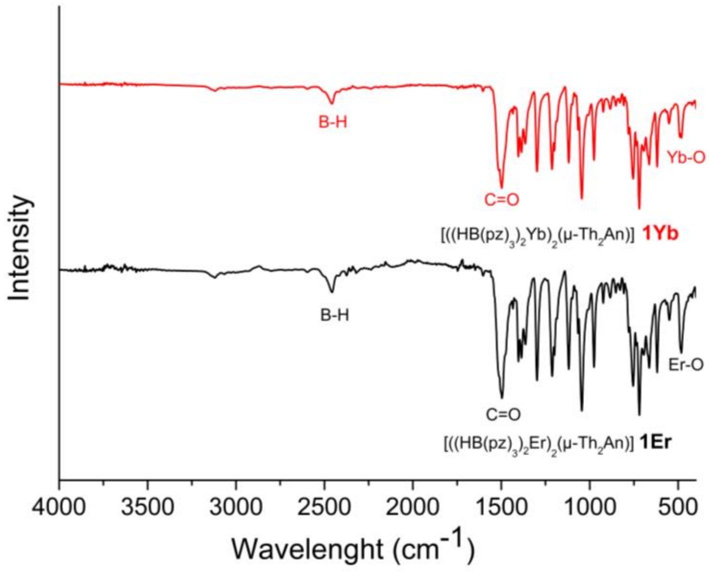


**Figure S1.** The ATR spectra of **1Yb** and **1Er** with the main bands highlighted.

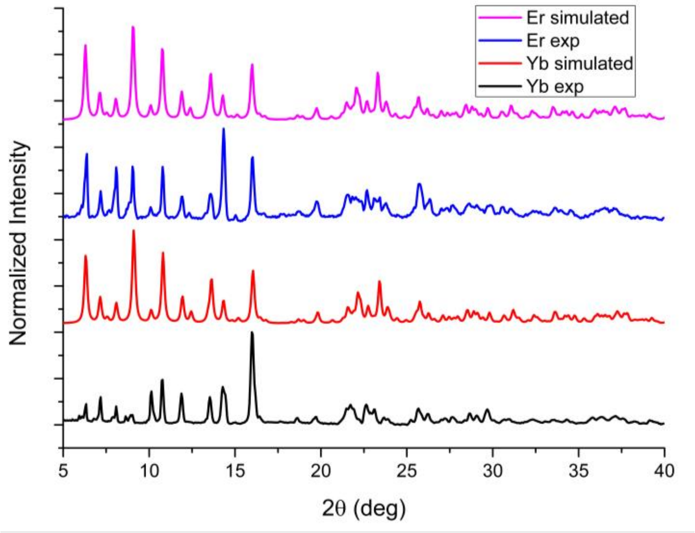


**Figure S2.** PXRD pattern for **1Yb** and **1Er**, experimental (exp) and simulated from crystal structure (simulated).

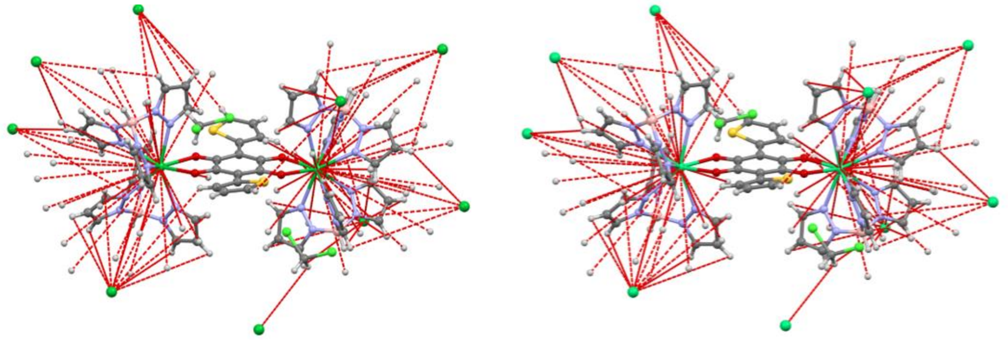

(a) (b)

**Figure S3.** Representation of the intermolecular $Ln^{III}$ ···H contact interactions in the 5-7.5 Å range for (a) **1Yb** and (b) **1Er**.

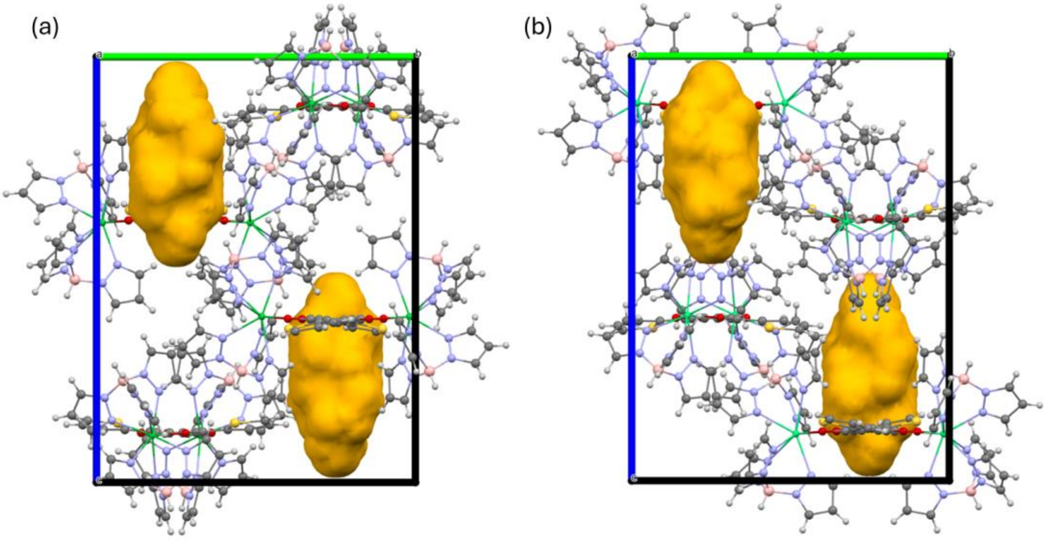


**Figure S4.** Contact surface area representation of the voids obtained through Mercury software for **1Yb** (a) and **1Er** (b).

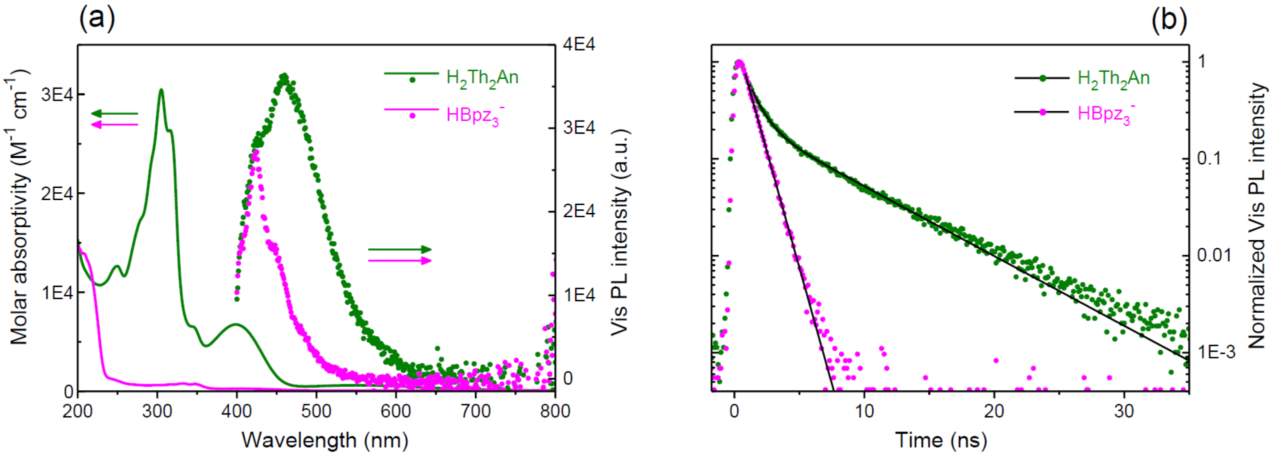


**Figure S5.** Optical spectroscopy of ligand species in diluted ACN solutions (excitation at 350 nm). (a) Molar absorptivity (left scale) and Cw Vis PL spectra (right scale); (b) Time-resolved Vis PL decay traces. Dots: Experimental data; Solid lines: Mono- and biexponential decay fits.
